# Supplementary material for: Predicting visual function by interpreting a neuronal wiring diagram
Source: Nature. 2024 Oct 2;634(8032):113–23. doi: 10.1038/s41586-024-07953-5 (PMC11446822; doi:10.1038/s41586-024-07953-5)
Supplement: Supplementary file 6 — CRF and ERF predictions for individual TmY4 and TmY9 cells. Analogous to Supplementary Data 3, but for TmY target types. Shown are the top four monosynaptic pathways, the strongest pathway passing through each of the top ten intermediary types (ranking from Extended Data Fig. 7), and the trisynaptic pathway Tm1–TmY–Dm3–TmY (see the section entitled Prediction of spatial normalization). [file 41586_2024_7953_MOESM6_ESM.zip › DataS4/TmY9qΓèÑ/720575940638437860.pdf]

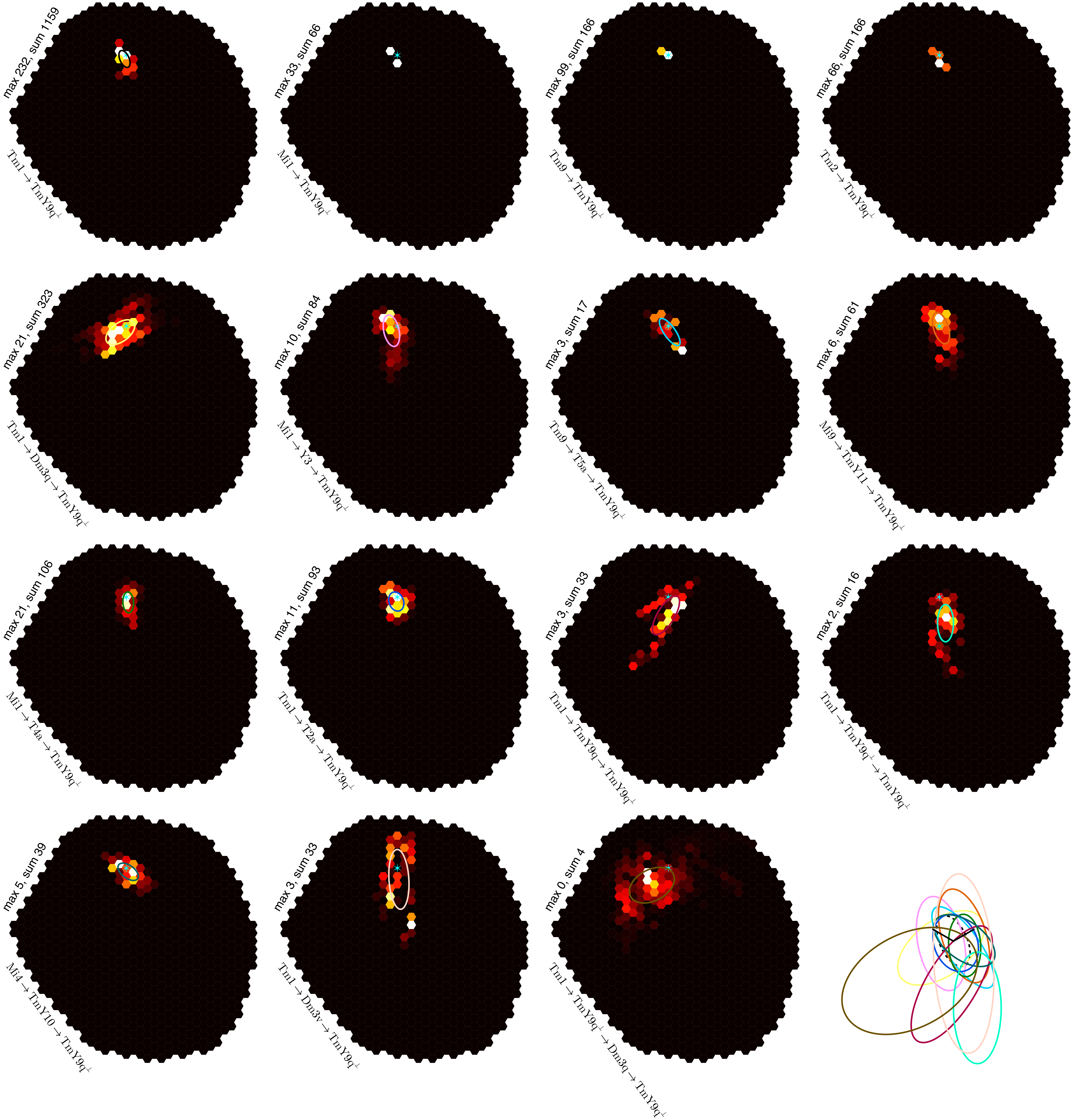
max 232, sum 1159
Tm1 → TmY9q⊥
max 33, sum 66
Mi1 → TmY9q⊥
max 99, sum 166
Tm9 → TmY9q⊥
max 66, sum 166
Tm2 → TmY9q⊥
max 21, sum 323
Tm1 → Dm3q → TmY9q⊥
max 10, sum 84
Mi1 → Y3 → TmY9q⊥
max 3, sum 17
Tm9 → T5a → TmY9q⊥
max 6, sum 61
Mi9 → TmY11 → TmY9q⊥
max 21, sum 106
Mi1 → T4a → TmY9q⊥
max 11, sum 93
Tm1 → T2a → TmY9q⊥
max 3, sum 33
Tm1 → TmY9q → TmY9q⊥
max 2, sum 16
Tm1 → TmY9q⊥ → TmY9q⊥
max 5, sum 39
Mi4 → TmY10 → TmY9q⊥
max 3, sum 33
Tm1 → Dm3v → TmY9q⊥
max 0, sum 4
Tm1 → TmY9q⊥ → Dm3q → TmY9q⊥
